# Overcoming the sorafenib resistance mechanism in FLT3-mutated acute myeloid leukemia: molecular basis and new targets

**DOI:** 10.3389/fonc.2025.1713174

**Published:** 2025-12-11

**Authors:** Hui Peng, Min Li, Yuan Yuan Peng, Xiao Lan Li, Jing Yang, Qiang Ge Sun, Kui Song

**Affiliations:** 1 Department of Hematology, The First Affiliated Hospital of Jishou University, Jishou, Hunan, China; 2 Medical College, Jishou University, Jishou, Hunan, China; 3 Department of Hematology, Affiliated Hospital of Guangdong Medical University, Zhanjiang, Guangdong, China

**Keywords:** FLT3 mutations, sorafenib resistance, AML, targeted therapy, FLT3 inhibitors

## Abstract

Acute myeloid leukemia (AML) originates from myeloid hematopoietic stem cells. Approximately 30% of patients exhibit FMS-like tyrosine kinase 3 (FLT3) mutations clinically, which is associated with a poor prognosis. FLT3 tyrosine kinase inhibitors (FLT3-TKIs), including sorafenib, demonstrate efficacy in FLT3-mutated AML, but resistance remains a significant challenge. However, various mechanisms have led to the rapid development of resistance to sorafenib treatment, including both primary and secondary drug resistance. Primary resistance refers to sorafenib’s initial treatment failure due to redundant signaling pathways and tumor heterogeneity, while secondary resistance develops after prolonged therapy through new genetic mutations or activation of alternative pathways. This study systematically examines mechanisms of sorafenib resistance in AML, including tumor genetic changes and the bone marrow microenvironment. It outlines classic mechanisms, such as FLT3 functions, kinase mutations, and cellular signaling pathways, while also addressing gaps in knowledge regarding resistance driven by metabolic factors and the bone marrow environment. Furthermore, the paper explores novel FLT3 inhibitors and combination therapies, while outlining future directions for precision intervention through dynamic monitoring of clonal evolution. This review provides a comprehensive framework for understanding and addressing sorafenib resistance, offering insights into future therapeutic strategies for FLT3-mutated AML.

## Graphical Abstract

**Figure:**
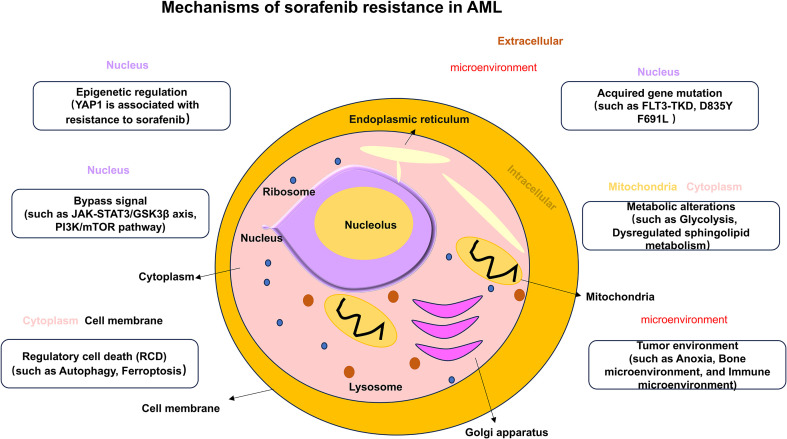

## 1 Introduction

The FMS-like tyrosine kinase 3 (FLT3) gene mutation is among the most prevalent genetic alterations in Acute myeloid leukemia (AML), occurring in approximately 30% of cases. The most frequent types are FLT3 internal tandem duplication (FLT3-ITD, 20–25%) and FLT3 tyrosine kinase domain (FLT3-TKD, 5–10%) (1). FLT3-ITD mutations are associated with a higher relapse rate, overall survival (OS) rate, and disease-free survival (DFS) are short, and the clinical efficacy is poor (2). The clinical prognosis of FLT3-TKD remains controversial. Meta-analysis by Li et al. demonstrated that FLT3-TKD improves DFS and OS in Asian AML patients, whereas it indicates a poorer prognosis for DFS in Caucasian AML patients. The co-mutation of FLT3 wild-type and Nucleophosmin 1 (NPM1) enhances both the risk of recurrence and overall survival (3). Additionally, high-load FLT3-ITD is frequently associated with adverse factors from unsuccessful induction chemotherapy (4).

FLT3 gene is primarily expressed in immature hematopoietic stem cells (HSCs) and is crucial for developing immune cells. When FLT3 binds to its FLT3 ligand (FLT3L), it induces FLT3 dimerization and activation through autophosphorylation at tyrosine residues, triggering important signaling pathways (PI3K/AKT, MAPK, JAK2/STAT5) (5) that regulate cell proliferation and inhibit apoptosis (6).

FLT3-mutated AML treatments have improved with targeted therapies like SOR, a type II multikinase inhibitor. It has demonstrated effectiveness in improving hematopoietic function and overall survival in relapsed FLT3-ITD AML, restoring hematopoietic function in 12 of 13 patients, and prolonging survival in relapsed/refractory (R/R) cases (7). However, long-term monotherapy or combination therapy increases the likelihood of drug resistance. Research indicates that sorafenib resistance is associated with molecular abnormalities, bypassed signaling pathways, metabolic changes, and alterations in the tumor microenvironment.

Research has expanded beyond small-molecule inhibitors to understand drug resistance mechanisms in FLT3-mutated AML and improve FLT3 inhibitor efficacy. These strategies include combining molecularly targeted agents, nanoparticle-loaded combination drugs, low-toxicity drug combinations, small-molecule inhibitor combinations, other combination therapies, and combinations with immunotherapeutic drugs (8).

## 2 Methods

To ensure a comprehensive overview of the current landscape on [Mechanisms of Sorafenib Resistance in FLT3-Mutated Acute Myeloid Leukemia and new targeted therapy], a systematic literature search was conducted. Data Sources: The PubMed databases were searched. Time Frame: The search was limited to articles published from the inception of each database until November 2025. Search Terms: Key search terms included “FLT3 mutations”, “sorafenib resistance”, AML, targeted therapy, and FLT3 inhibitors. These terms were combined using Boolean operators (AND, OR). Study Selection: Articles were selected based on their relevance to the topic of [Mechanisms of Sorafenib Resistance in FLT3-Mutated Acute Myeloid Leukemia]. Only original research and review articles published in English or Chinese were considered for inclusion.

## 3 The structural function of FLT3 and the mechanism of action and clinical progress of sorafenib

### 3.1 Structure, expression of FLT3, and function

*FLT3*, located on chromosome 13q12, consists of 24 exons (1)and encodes a membrane-bound glycosylated protein and a non-glycosylated isomer. FLT3 is a crucial gene involved in cellular signaling, sharing similarities with other receptor tyrosine kinases, FMS-like tyrosine kinase 3 (FMS), Platelet-derived growth factor receptor (PDGFR), and the stem-cell factor receptor (CD117, KIT) (9). FLT3 is primarily expressed in human bone marrow, particularly stem cells, progenitor cells (10), and dendritic cell precursors (11). The ligand FLT3 (FL) is produced in gonads and hematopoietic tissues by the bone marrow stroma (12).

**Figure 1:**
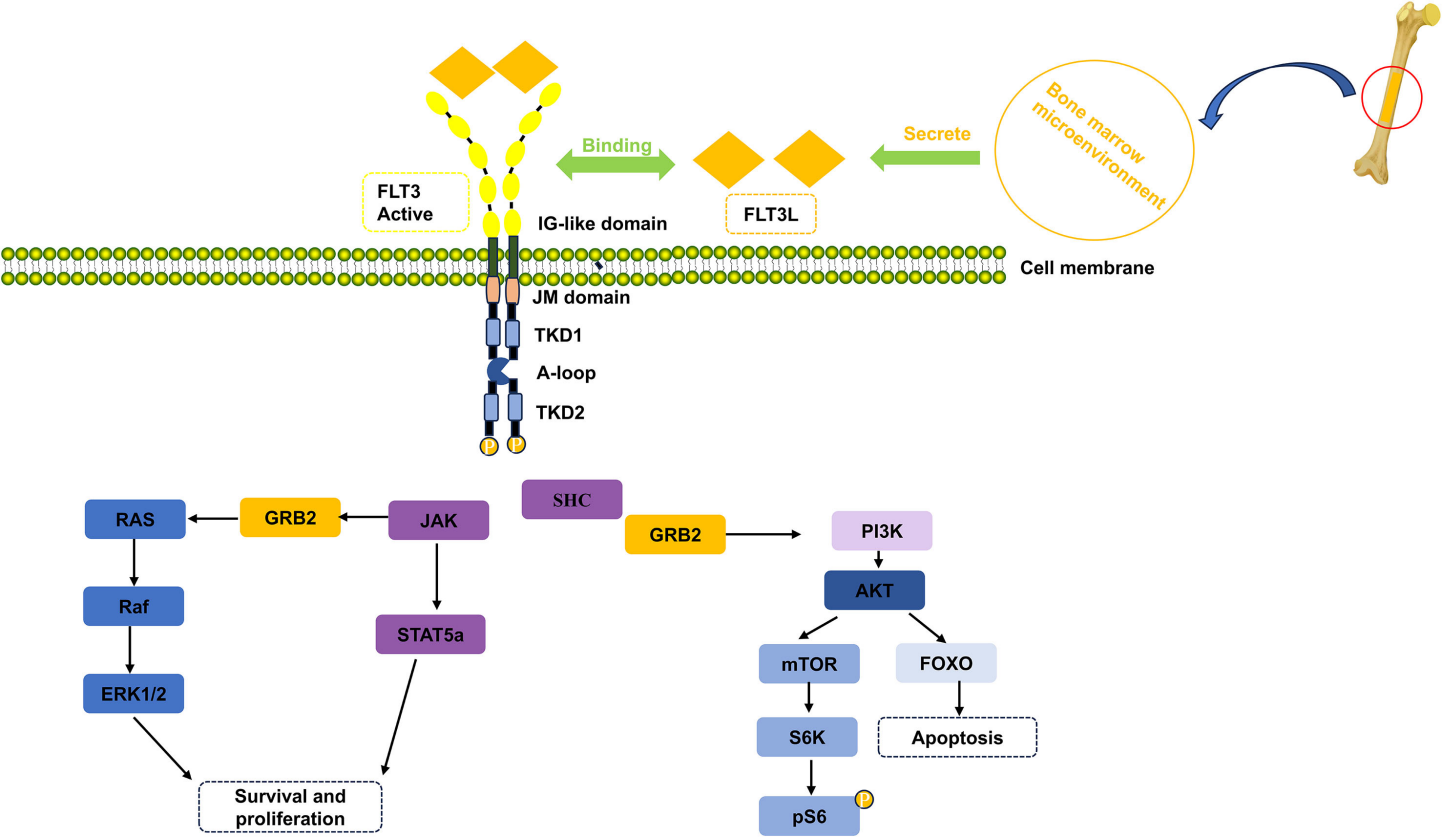
Structure of FLT3-mutated receptor and FLT3 signaling pathways (13). After FLT3L binds to the activated FLT3, it activates the survival, proliferation, and apoptosis pathways. FLT3L, Fms-related tyrosine kinase 3 ligand; FLT3, Fms-like tyrosine kinase; IG-like-domain, IG-like domain-containing proteins; JM domain, juxtamembrane domain; TKD1, tyrosine kinase domain 1; A-loop, activation loop; TKD2, tyrosine kinase domain 2; JAK, janus kinase; STAT5a, Signal Transducer And Activator Of Transcription 5A; GRB2, Growth Factor Receptor Bound Protein 2; RAS, oncogene; Raf, Raf-1 Proto-Oncogene, Serine/Threonine Kinase; ERK1/2, Extracellular regulated protein kinases 1/2; SHC, SHC Adaptor Protein 1; PI3K, Phosphatidylinositol 3-kinase; AKT, Protein kinase B; mTOR, mammalian target of rapamycin; FOXO, Forkhead Box Protein; S6K, Ribosomal Protein S6 Kinase B1; pS6, Phospho-S6 Ribosomal Protein. Created in ScienceSlides.

FLT3 is mainly composed of four regions: an extracellular domain containing five immunoglobulin-like subdomains, a transmembrane domain, an intracellular juxtamembrane (JM) domain, and an intracellular C-terminal domain, including two tyrosine kinase subdomains. Tyrosine kinase domain (TKD1) and Tyrosine kinase domain (TKD2) are connected by an activation loop (A-loop) (1). (**Figure 1**) FLT3 exists as a monomer in its inactive configuration, but mutations such as FLT3-ITD leads to spontaneous dimerization by disrupting inhibitory interactions. By binding to FLT3, FLT3L triggers multiple signaling cascades, including Ras/Raf and PI3K (14), influencing key transduction and adaptor proteins (**Figure 1**). This binding results in cell proliferation, decreases cell death, and prevents cell maturation, underscoring the substantial effect of FLT3L and FLT3 on cellular development.

### 3.2 FLT3 mutation and proliferative activation of AML

In 1996, activating mutations in FLT3 receptor were first identified in AML (15). These mutations fall into ITD and TKD. ITD mutations are the most common type of FLT3, mainly located in or near the proximal domain of the receptor. Mutations in ITD result in dimerization, constitutive phosphorylation, and activation of kinase domains in FLT3 receptor, which are no longer ligand-dependent, promoting AML cell proliferation (16). TKD mutations are due to missense point mutations, occurring in the activation loop (ring A) of the kinase domain, replacing a single amino acid, primarily involving aspartic acid 835 in the kinase domain (17) and amino acid residues such as D835 and Y842. In addition, a few mutations located at the N676 and F691 residues are also observed. When FLT3 is in an inactive state, the A loop blocks ATP and substrates from entering the kinase domain. The substitution of those amino acid residues mentioned earlier will affect the inhibitory effect of the A loop, leading to kinase activation and downward signal transmission (12).

### 3.3 The mechanism of action and clinical progress of sorafenib

Given FLT3’s central role in leukemogenesis, it has become a prime target for therapeutic intervention.

Sorafenib is an oral first-generation type II inhibitor targeting important kinases such as RAF, VEGFR, PDGFR, KIT, and RET. (18, 19). In treating FLT3-ITD-positive (FLT3-ITD+), sorafenib inhibits the abnormal activation of downstream signaling pathways by competitively binding to the ATP-binding site of FLT3 kinase domain and blocking its phosphorylation process. In the long-term follow-up of sorafenib clinical trials, patients under 60 years old newly diagnosed with AML who received sorafenib combined with standard chemotherapy and maintenance therapy showed a 5-year event-free survival (EFS) rate of 41:27% (hazard ratio *HR* 0.68; *p* = 0.011) and a 5-year relapse-free survival (RFS) rate of 53:36% (*HR* 0.64; *p* = 0.035). Among the 88% of patients who received allogeneic hematopoietic stem cell transplantation (SCT) for relapsed disease, the 4-year relapse rate was 54:35%, and the overall survival rate was 32:50%. The data demonstrate that chemotherapy combined with sorafenib not only significantly prolonged event-free survival (EFS) and relapse-free survival (RFS), but also achieved a statistically significant overall survival (OS) extension, confirming the drug’s efficacy in leukemia treatment (20). However, sorafenib monotherapy shows limited effectiveness, achieving a Complete response (CR) of only 10% in FLT3-ITD patients (21). Combination therapies, particularly with hypomethylating agents (HAM) and the combination of cladribine, high-dose cytarabine, granulocyte colony-stimulating factor, and mitoxantrone (CLAG-M), have shown better outcomes in FLT3-mutated AML (22). Achieving desired outcomes with sorafenib monotherapy in FLT3-mutated AML patients is challenging due to disease variability. Therefore, combination treatment is essential to enhance sorafenib’s efficacy.

## 4 Mechanism of sorafenib resistance in AML

**Figure 2:**
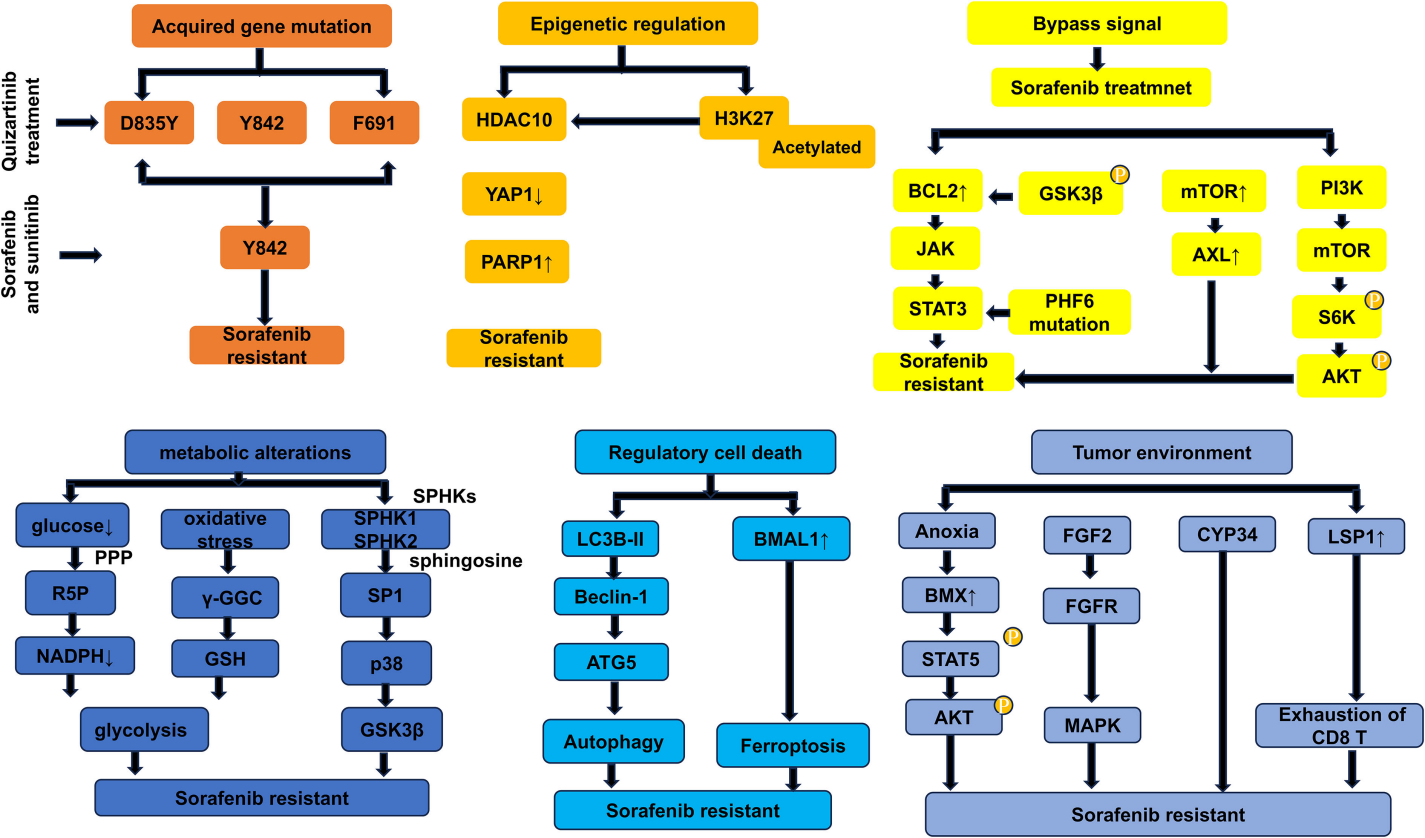
General diagram of the resistance mechanism of Sorafenib. HDAC10, histone deacetylase 10; YAP1, Yes1 Associated Transcriptional Regulator; HDK27, histone H3 lysine 27; PARP1, Poly (ADP-Ribose) Polymerase 1; BCL2, B-cell lymphoma-2; GSK3β, glycogen synthase kinase 3beta; JAK, janus kinase; STAT3, signal transducer and activator of transcription 3; PH6, homeodomain finger protein 6; mTOR, mammalian target of rapamycin; AXL, AXL Receptor Tyrosine Kinase; PI3K, Phosphatidylinositol 3-kinase; S6K, Ribosomal Protein S6 Kinase B1; AKT, Protein kinase B; PPP, pentose phosphate pathway; R5P, ribulose-5-phosphate; γ-GGC, γ-Glu-Cys; GSH, synthesis of glutathione; Sphingosine kinases, SPHKs; SPHK1, Sphingosine kinases 1; SPHK2, Sphingosine kinases 2; SP1, sphingosine-1-phosphate; p38/MAPK, P38 mitogen-activated protein kinase; LC3B-II, Microtubule-associated protein 1 light chain 3; BMAL1, Basic Helix-Loop-Helix ARNT Like 1; BMX, Bone marrow kinase on chromosome X; STAT5, Signal Transducer and Activator of Transcription 5; FGF2, fibroblast growth factor 2; FGFR, FGF receptor; CYP34, cytochrome P450 enzymes; LSP1, Lymphocyte-specific protein 1; CD8, Cytotoxic T lymphocyte.

AML is characterized by pronounced genetic, epigenetic, and metabolic heterogeneity, which underlies the high risk of therapeutic resistance. The heterogeneity of FLT3-ITD mutations in AML patients exacerbates disease refractoriness and increases the likelihood of resistance to FLT3 inhibitors. The mechanisms underlying sorafenib resistance are multifaceted: acquired gene mutation, epigenetic regulation, activating bypass signals, metabolic alterations, regulatory cell death, and the tumor microenvironment. Please refer to (**Figure 2**, **Table 1**) for details on the drug resistance mechanism.

**Figure 3:**
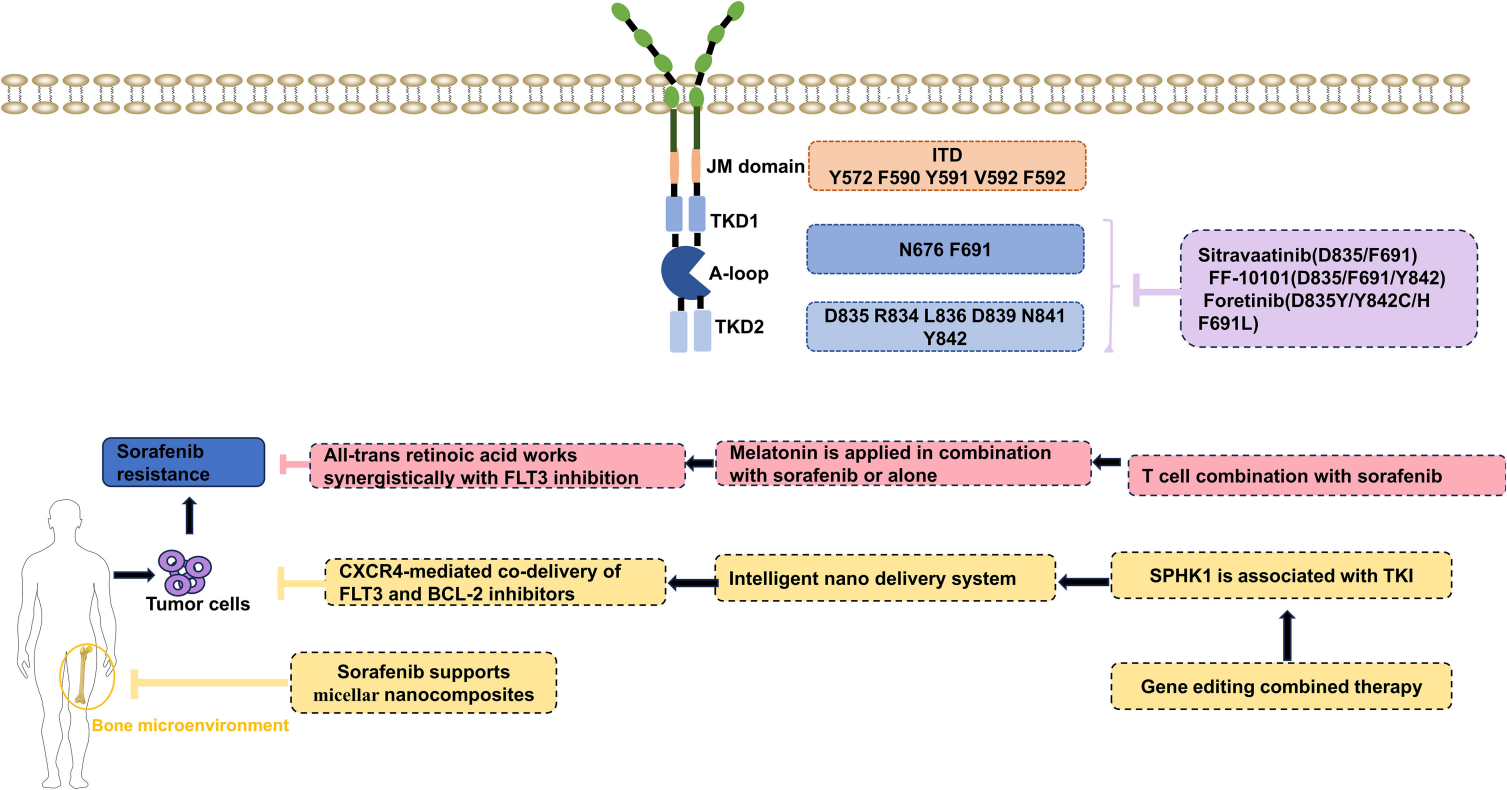
Target therapies and overcome the sorafenib resistance. JM domain, juxtamembrane domain, TKD1, tyrosine kinase domain 1; A-loop,activation loop; TKD2, tyrosine kinase domain 2; CXCR4, C-X-C chemokine receptor type 4; FLT3, Fms-like tyrosine kinase; SPHK1, Sphingosine Kinase 1; TKI, Tyrosine kinase inhibitors. Created in ScienceSlides.

**Table 1:** The resistance mechanism of sorafenib in AML.

Classification of drug resistance mechanisms	Key molecule/pathways	Functional effect
Acquired gene mutation	D835Y	The combination with type II inhibition is complex and prone to drug resistance.
	F691	It was endowed with resistance to type I and type II inhibitors.
	A848P	Secondary resistance to sorafenib and sunitinib.
Epigenetic regulation	YAP1	Deacetylation of YAP1 promotes the resistance to Chemo- and Targeted Therapy in FLT3-ITD+ AML Cells.
Bypass signal	JAK-STAT3/GSK3β axis	JAK-STAT3/GSK3β axis is associated with the proliferation, survival, and apoptosis signaling pathways and promotes sorafenib resistance.
	PI3K/mTOR pathway	In drug-resistant cells, this pathway is activated, leading to cell proliferation.
Metabolic regulation	GSH	Increased metabolic demand reduces the entry of glucose into the pentose phosphate pathway (PPP), while oxidative stress may promote glutathione synthesis.
	SPHK1	SPHK1 overexpression enhances AML cell resistance to sorafenib by elevating β-catenin levels.
Regulatory cell death	LC3B-II, Beclin-1, and ATG5, p62 degradation	Activate autophagy and promote resistance.
	BMAL1	The increase in BMAL1 levels promotes sorafenib resistance.
Tumor environment	BMX	BMX activates the STAT5 signaling pathway, promoting the proliferation of AML cells.
	FGF2, CYP34	Stromal cells and FL promote drug resistance.
	LSP1	LSP1 is closely related to resistance to sorafenib.

HDAC10, Histone Deacetylase 10; YAP1, Yes1 Associated Transcriptional Regulator; JAK, Janus Kinase; STAT3, signal transducer and activator of transcription 3; GSK3β, glycogen synthase kinase 3beta; FLT3, Fms-like tyrosine kinase; FLT3-ITD, FLT3 internal tandem; HK2, hexokinase 2; AKT, Protein kinase B; SPHK1, Sphingosine Kinase 1; p62, Sequestosome 1; β-catenin, Catenin beta-1; LC3B-II/I, Microtubule-associated protein 1 light chain 3; ATG5, Autophagy Related 5; Basic Helix-Loop-Helix ARNT Like 1 (BMAL1), Arabidopsis β-amylase; STAT5, signal transducer and activator of transcription; P450, Cytochrome P450; BMX, BMX Non-Receptor Tyrosine Kinase; LSP1, Lymphocyte Specific Protein 1; CD8, Cytotoxic Tlymphocyte.

### 4.1 Acquired gene mutation

Patients receiving long-term FLT3 inhibitors are prone to developing secondary resistance, involving both FLT3 mutations and non-FLT3 functional factors (23). For instance, FLT3-mutated patients treated with quizartinib may develop secondary mutations at the D835Y residue and Y842, as well as the F691 “gatekeeper” mutation. The D835Y mutation occurs at the activation loop, causing conformational changes that maintain the loop’s open state. This exposes the kinase active site, continuously activating downstream proliferative signaling pathways and leading to resistance to type II inhibitors (24). Similarly, F691L and other gatekeeper mutations impair the binding site, reducing the efficacy of FLT3 inhibitors like quizartinib and ultimately resulting in resistance (25). Changes in the ATP-binding pocket of active kinases, like FIPL1-PDGFRα (26), KIT (27), EGFR (28), and EML4-ALK (29). New FLT3-TKD mutations have been identified in relapsed patients treated with type II inhibitors like quizartinib and sorafenib, reducing their efficacy. Mutations at F691, the gatekeeper residue of the TK domain, confer resistance to both type I and type II inhibitors (30). A study identified additional mutations in IDH1/IDH2, TP53, FLT3-N676K, and FLT3-N841K among patients on FLT3 II inhibitors (31). Relevant studies have demonstrated that secondary resistance to sorafenib and sunitinib is also associated with mutations in A848P (32). Recent research found novel mutations in gilteritinib-resistant cells, including MYCN D31P and C695F. MYCN is an important transcription factor in cancer. The C695F mutation may affect drug binding and contribute to resistance. While the D31P mutation lowers transcriptional activity, the C695F mutation can increase MYCN stability, leading to a buildup of non-functional MYCN (33). Targeting these mutations could enhance AML therapy.

### 4.2 Epigenetic regulation

YAP1 (Yes-associated protein 1) is a transcriptional regulator involved in cancer progression, acting as both an oncogene and a tumor suppressor. In leukemia, particularly with FLT3-ITD mutations, YAP1 levels are reduced, and restoring its expression can delay tumor progression. The inactivation of histone deacetylase (HDAC) influences gene expression and tumorigenesis. Previous studies have identified that histone deacetylase 3 (HDAC3) enhances the DNA damage repair by activating AKT and preventing leukemia cells from chemotoxicity. Research showed low YAP1 in sorafenib-resistant cell lines (MV4-11-SorR and MOLM13-SorR), with upregulation promoting drug resistance via PARP1. Conversely, the siRNA-mediated knockdown of YAP1 and PAR1 inhibitors increases drug sensitivity. Furthermore, to investigate whether YAP1 was deacetylated by HDAC10 (histone deacetylase 10, HDAC10), MV-4–11 cells were treated with Chidamide. The results showed that low HDAC10 expression led to increased YAP1 expression and decreased PARP1 expression, with western blot analysis revealing elevated histone H3 lysine 27(H3K27) acetylation levels (34). Hence, HDAC10 is acetylated by histone H3 lysine 27(H3K27), resulting in decreased levels of YAP1 and resistance, suggesting that boosting YAP1 expression could offer therapeutic potential.

### 4.3 Bypass signal and sorafenib resistance

#### 4.3.1 JAK-STAT3/GSK3β axis

Acquired mutations in FLT3-ITD and FLT3-TKD activate proliferation and survival pathways, contributing to sorafenib resistance (35). After sorafenib treatment, BCL2 upregulation in leukemia cells was linked to JAK/STAT3 signaling, which promotes sorafenib resistance (36). Additionally, FLT3-ITD-positive patients with mutations in suppressor mutations showed poor responses to sorafenib. TP53 mutations may increase resistance by activating STAT3. While PHF6 mutations can enhance JAK/STAT signaling, contributing to this effect (37). Phosphorylated GSK3β is also involved (38).KEGG analysis indicated significant enrichment in apoptosis and signaling pathways, with increased mTOR and Axl levels suggesting their roles in sorafenib resistance (39).

#### 4.3.2 PI3K/mTOR pathway

The PI3K-mTOR signaling pathway is important for cell growth, survival, and metabolism, and is often dysregulated in diseases like hematopoietic malignancies. Activating mutations in FLT3, NRAS, KRAS, and KIT (a regulatory subunit of PI3K), as well as functional impairments in PTEN, can lead to abnormal activation of this pathway (40). Lindblad et al. found that resistant cell lines often exhibited upregulation of genes associated with cell survival and proliferation. Lindblad et al. (41) observed mTOR and AKT pathway enrichment in MLOM-13 and MV4–11 drug-resistant cells (sorafenib-resistant cells). Furthermore, selective phosphorylation of mTOR substrates S6K and AKT was also detected in the phosphoprotein antibody cohort. This indicates that PI3K/mTOR activation leads to abnormal expression of survival proteins, thereby promoting resistance to multiple FLT3 inhibitors in these drug-resistant cells. The findings suggest that targeting the PI3K/mTOR pathway may be an effective treatment strategy for overcoming resistance to FLT3 inhibitors in AML.

### 4.4 Resistance of sorafenib to metabolic alterations

#### 4.4.1 glycolysis

Previous studies have demonstrated that the constitutive activation of FLT3-ITD reduces mitochondrial respiration and promotes glycolysis, contributing to the Warburg effect in cancer (42). In FLT3-ITD-positive leukemia, these metabolic changes led to resistance, marked by mitochondrial dysfunction and increased glycolysis (42), which supported tumor growth. Through stable isotope tracing experiments, You et al. (43) revealed that glucose carbon in drug-resistant cells (BaF3/ITD and MV-4-11-R) primarily enters the glycolytic pathway, characterized by increased lactic acid secretion and accumulation of glycolytic intermediates. These cells exhibit enhanced glucose dependence, with exogenous glucose deprivation significantly inhibiting their proliferative capacity. Metabolomic analysis further demonstrated glycolytic enzymes (e.g., hexokinase). Studies indicated that increased metabolic demands reduce glucose entry into the phosphopentose pathway (PPP), while oxidative stress may enhance glutathione synthesis. Isotope tracing confirmed significantly reduced glucose flux into the PPP in drug-resistant cells, manifested by decreased ribulose-5-phosphate (R5P) and NADPH production. The oxidative arm of PPP provides NADPH for glutathione regeneration, maintaining redox balance. Drug-resistant cells showed markedly elevated GSH levels and GSH/GSSG ratios, accompanied by upregulated GSH-synthesizing enzymes (GCLC, GSS, GPX). Metabolomic analysis revealed accumulation of γ-glutathione-cysteine (γ-Glu-Cys, GSH precursor), suggesting activation of the GSH biosynthetic pathway. Additionally, transcription factor Nrf2 is upregulated in drug-resistant cells, enhancing cellular resistance to sorafenib-induced oxidative stress by regulating antioxidant enzymes (GCLC, GPX, SOD). Modifying tumor glycolysis is emerging as a promising strategy in AML treatment.

#### 4.4.2 Dysregulated sphingolipid metabolism

Sphingosine kinases (SPHKs), particularly Sphingosine kinase 1 (SPHK1) and Sphingosine kinase 1 (SPHK2), are important enzymes in sphingolipid metabolism, converting sphingosine to sphingosine-1-phosphate (S1P) (44). SPHK1 activation and subsequent S1P production are closely associated with cancer prognosis and adverse reactions (45). Studies have shown that the SPHK/S1P signal pathway activates the p38-GSK3β-β-catenin pathway, promoting osteoblast formation with elevated S1P2 expression in relapsed AML patients. SPHK1 overexpression helps AML cells resist sorafenib by enhancing β-catenin levels (46), suggesting that targeting the SPHK1/S1P axis and β-catenin could mitigate sorafenib resistance.

### 4.5 Regulatory cell death and sorafenib resistance in AML

#### 4.5.1 Autophagy

Autophagy is implicated in cellular metabolism by degrading abnormal substances and recycling materials for new molecule synthesis, thereby maintaining homeostasis and enabling stress responses. Recent studies link altered autophagy to cancer development and treatment resistance, particularly leukemia (47–49). The process is mainly regulated by mTOR signaling and involves proteins like ATG8 and the ATG12/ATG5/ATG16L1 complex, which are vital for converting LC3-I to LC3-II, a key autophagy marker. In Baf3 sorafenib-resistant cells with FLT3-D835Y or FLT3-ITD+D835Y mutations, autophagy markers (LC3B, Beclin-1, ATG5, and p62) were detected. Compared to sorafenib-sensitive Ba/F3-ITD mutant cells, Baf3 cells with FLT3-D835Y or FLT3-ITD+D835Y mutations exhibited higher expression of LC3B-II, Beclin-1, and ATG5, along with reduced p62 degradation (50). The study revealed that sorafenib-resistant cells showed increased LC3B-II/I ratios and ATG5 expression, corresponding to decreased p62 levels. This suggests that sorafenib-resistant FLT3-ITD-positive cell lines may also exhibit autophagy overexpression, where protective autophagy is activated by acquired resistance mutations. Targeting autophagy can potentially reverse this resistance.

#### 4.5.2 Ferroptosis

Ferroptosis, a programmed cell death mechanism primarily mediated by iron and reactive oxygen species (ROS), was first identified in 2012 (51). It involves lipid peroxidation caused by glutathione depletion and reduced activity of glutathione peroxidase 4 (GPX4), which increases ROS levels (48). Key regulators include System Xc-(composed of SLC3A2 and SLC7A11), glutathione, and GPX4. This mechanism is associated with a variety of diseases, including tumors (52), diabetes (53), and neurodegenerative disorders (54). SLC7A11 serves as the primary subunit of System Xc-, implicated in the cellular uptake of cystine, which is vital for the synthesis of glutathione (GSH). The downregulation of p53 reduces SLC7A11, impairing cystine uptake (55) and lowering GPX4 levels, increasing susceptibility to ferroptosis.

Autophagy contributes to the degradation of ferroptosis inhibitors such as ferritin and Basic Helix-Loop-Helix ARNT Like 1 (BMAL1) (56). BMAL1 is a core transcription factor that binds to E-box elements within the promoter region. Autophagy interacts with the CLOCK protein heterodimer, further influencing cellular processes. Zheng et al. confirmed the involvement of BMAL1 in the progression of ferroptosis in AML; its downregulation enhances sensitivity to ferroptosis and chemotherapy, while upregulation provides resistance. Zheng et al. (57) demonstrated that BMAL1-overexpressing AML cells developed resistance to dasatinib, venetoclax, and sorafenib. This indicates that BMAL1 plays a pivotal role in ferroptosis in AML: downregulation of BMAL1 enhances sensitivity to ferroptosis and chemotherapy, while upregulation increases resistance. The findings reveal that elevated BMAL1 levels rendered AML cells resistant to sorafenib dasatinib, and venetoclax. Therefore, inducing ferroptosis may be a promising therapeutic strategy in this context.

### 4.6 Tumor environment and sorafenib resistance in AML

#### 4.6.1 Anoxia

Stromal and endothelial cells in bone marrow are essential for the survival and drug response of AML patients (58). Bone marrow kinase on chromosome X (BMX) is highly expressed in these cells, promoting tumor cell proliferation and linking to chemotherapy resistance. Research indicated that BMX levels increase in conditions like clear renal cell carcinoma (59)and under hypoxia, which elevates HIFs and VHL gene mutations. An RNA-Seq analysis revealed that sorafenib resistance heightened bone marrow hypoxia, increasing BMX expression. This upregulation is also seen in FLT3-ITD-positive murine models. To investigate the BMX-mediated resistance mechanism of sorafenib in BMX, Oosterwijk et al. (60)overexpressed various BMX variants in HEK293 cells and treated MOLM13 cells with sorafenib and found that sorafenib mediated the phosphorylation of STAT5 and AKT. Conversely, knocking down BMX in MV-4–11 cells inhibited sorafenib-mediated STAT5 phosphorylation. Hence, BMX activates the STAT5 signaling pathway, which promotes an alternative survival strategy. Therefore, targeting hypoxia may be a promising strategy to improve treatment outcomes.

#### 4.6.2 Bone microenvironment

In 1978, Schofield proposed the bone marrow microenvironment (BMM) (61), highlighting its role in the human hematopoietic system. BMM, consisting of stromal cells and molecules, significantly impacts Hematopoietic stem cells (HSCs). To investigate whether FGF2 (fibroblast growth factor 2) protects FLT3-ITD AML cells by activating the FGF receptor (FGFR), Traer et al. (62) introduced the FGFR inhibitor PD173074 into MLOM14 cells that already contained FGF2 and AC220. This significantly reduced FGF2’s protective effect. FGF2-mediated drug resistance was further enhanced by activating FGFR1 and downstream MAPK signaling factors.

Studies indicated that the bone marrow microenvironment expresses various cytochrome P450 enzymes (CYPs), which appear to mediate the local metabolism of endogenous factors such as retinoids and chemotherapeutic agents. CYP34, in particular, is predominantly involved in the hepatic metabolism of numerous drugs and may provide a protective chemical shield for FLT3-AML and multiple myeloma within the bone marrow microenvironment (63). Chang et al. (64) demonstrated that CYPA34 protects FLT3-ITD AML cells in Bone Marrow Mesenchymal Stem Cells (BMSCs). They cultured AML cells with BMSCs and treated them with CYP3A4 knockdown (shCYP3A4) or control (pGIPZ empty lentiviral vector). Subsequently, the addition of sorafenib, quizartinib, and gefitinib at their half-inhibitory concentrations, which significantly inhibited AML cell proliferation. This confirmed that BMSC-mediated drug resistance was eliminated mainly by CYP3A4 knockdown in BMSCs.

#### 4.6.3 Immune microenvironment

The tumor immune microenvironment is characterized by a state of complex, dynamic regulation (65). The progression of AML is not only affected by itself but also by specific immunosuppressive cells in the body, such as tumor-associated macrophages (66), the upregulation of inhibitory regulatory T cells (67), and immune checkpoint molecules (68), which ultimately promote immune escape. Lymphocyte-specific protein 1 (LSP1) supports the immune system (69). It is highly expressed in lymphocytes, neutrophils, monocytes, and macrophages, and interacts with lymphocyte migration, activation, and intercellular interactions (70). Studies have shown that LSP1 is closely related to the progression of AML (71). Xu et al. found, based on the analysis of TCGA data, that the IC50 of sorafenib in the high LSP1 group was significantly lower than that in the low LSP1 group, indicating that LSP1 in AML patients was significantly negatively correlated with sorafenib sensitivity. LSP1 in cell lines with knockdown of FLT3 mutations was constructed by lentiviral infection. It was found that LSP1 was significantly negatively correlated with the prognosis of AML. Moreover, further analysis of single cells revealed that LSP1 was mainly expressed in malignant cells. AML cells with high expression of LSP1 could promote the exhaustion of CD8 T effector cells, thereby increasing the risk of sorafenib resistance (72). This suggests that LSP1 may support immune escape, an important factor in AML progression and treatment resistance.

## 5 Current treatment status and limitations, innovative diagnostic technologies and latest clinical translation current treatment status and limitations

### 5.1 FLT3 inhibitors in clinical trials and development

The occurrence of resistance to FLT3 inhibitors, such as Sorafenib, has accelerated the clinical treatment progress. The development of FLT3 inhibitors aims to improve the poor prognosis of FLT3-mutated acute myeloid leukemia (AML) (16). These drugs can be classified based on their specificity and mechanisms of action: from a specificity perspective, they are divided into first-generation and second-generation inhibitors (16, 73); from a mechanism perspective, they are categorized as Type I and Type II inhibitors. Type I inhibitors target activated FLT3 by binding to the ATP-binding site, with typical representatives including larstatinib, sunitinib, midostinib, renuvatinib, and gilteritinib. Type II inhibitors act on non-activated FLT3 by binding to hydrophobic regions near the ATP-binding site (74), with examples like tandostinib, sorafenib, and quinacitineib. By targeting different conformational states of the protein, these drugs provide diverse therapeutic options for patients with various FLT3 mutations. Additionally, **Table 2** summarizes clinically approved FLT3 inhibitors and new FLT3 inhibitors still in clinical development.

**Table 2:** Clinically approved drugs and preclinical drugs.

Name of the inhibitor	Generation/Type	Clinical stage/approval status	Applicable population	Main target	Response	The main drug resistance mechanism
Midostaurin (PKC412)	First/I	FDA approval, 2017	C-KIT/FLT3 mutated t (8;26)	FLT3, KIT, PDGFR	No results posted NCT01830361	Secondary mutations of FLT3-TKD (such as D835Y), bypass activation (RAS/MAPK)
Gilteritinib (ASP2215)	Next/I	FDA approval, 2018	R/R FLT3-ITD AML patients	FLT3(ITD/TKD), AXL	In the Phase III trial (NCT02421939), the median survival time of Gilteritinib was significantly longer than that of the salvage chemotherapy group (9.3 months vs 5.6 months)	FLT3-F691L gatekeeper mutation, AXL overexpression, RAS mutation
Quizartinib (AC220)	Next/II	Only in Japan, 2019	R/R FLT3-ITD AML	FLT3-ITD	QuANTUM-R(NCT02039726) trial: Median OS 6.2 months vs. 4.7 months (chemotherapy)	FLT3-TKD mutations (such as D835V/Y), bypass activation
Crenolanib (CP-868, 596)	Next/I	Phase II trial	FLT3-mutated AML	FLT3 (ITD/TKD), PDGFR	Phase II (NCT01657682) : The CR/Cri of patients who did not use FLT3 TKI was 23.1%, and that of those who had previously used FLT3 TKI was 13.3%	Bypass activation (such as STAT5), FLT3 is independent
Sorafenib (BAY 43-9006)	First/II	Phase II trial	R/R FLT3-ITD AML	FLT3, VEGFR, PDGFR, RAF	No results posted (NCT03622541)	Improve FLT3-TKD mutations and activate the RAF/MEK pathway.
FF-10101	Third/I	Phase I/II clinical trials	R/R FLT3-mutated AML	FLT3(Irreversible Binding	No results posted NCT03194685	FLT3 mutation and F691 mutation
Pexidartinib (PLX-3397)	First/II	Phase I/II	R/R AML	F691, FLT3-ITD	Phase I/II (NCT00783653): The overall response rate of the 90 patients was 21%.)	Similar to other inhibitors, it may involve new mutations or bypasses
Sunitinib (SU11248)	First/II	Phase I/II clinical trial	Leukemia patients over 60 years old with FLT3 mutations	FLT3, KIT, VEGFR, PDGFR	Phase I/II (NCT00783653): Some patients experience brief remission, with a low remission rate and short duration	FLT3-TKD mutation, bypass activation

ITD, internal tandem duplication; TKD, tyrosine kinase domain; CR/CRi, Complete Remission/Complete Remission with Incomplete Hematologic Recovery; FLT3 TKI, FLT3 tyrosine kinase inhibitors; STAT5, Signal Transducer and Activator of Transcription 5; R/R, relapsed/refractory; FLT3, FMS-like tyrosine kinase 3; VEGFR, Vascular endothelial growth factor receptor 2; PDGFR, Platelet-Derived Growth Factor; RAF, Raf-1 Proto-Oncogene Serine/Threonine Kinase; MEK, Mitogen-activated extracellular signal-regulated kinase.

### 5.2 Current limitations

At present, some FLT3 inhibitors have been approved in clinical practice. Relevant studies have shown that in the treatment of FLT3-ITD-positive AML patients, chemotherapy combined with FLT3 inhibitors, such as midostaurin combined with chemotherapy for newly diagnosed patients, improves overall survival (OS) (75), the efficacy bottleneck is significant. Studies have shown that during recurrence, secondary mutations (such as D835Y/V) often occur in FLT3 kinase domain. It activates downstream bypass pathways such as MAPK and PI3K/AKT, leading to treatment failure (76). On the other hand, the treatment-related toxicity is high, and patients have poor tolerance. Especially for elderly patients, treatment is often interrupted due to their inability to tolerate high-intensity chemotherapy and the side effects of the drugs (77). In the combined use of sorafenib with traditional cytotoxic induction and consolidation chemotherapy, it failed to effectively improve the overall survival of AML with FLT3 mutations (78). Moreover, there is an increase in the level of FLT3L, which further weakens the efficacy of the inhibitor (79).

In clinical practice, when patients show poor response or tolerance to FLT3 inhibitors, allogeneic hematopoietic stem cell transplantation (allo-HSCT) is typically recommended. Allo-HSCT is currently the only method that may cure FLT3-ITD AML, relying on the graft-versus-leukemia (GVL) effect of the donor’s immune system to clear tumor cells. However, there are numerous limiting factors for its application. The sources of suitable donors are limited, and only some patients can find HLA-matched donors. Transplantation-related complications are severe. Acute and chronic graft-versus-host disease (GVHD), infections, etc., significantly increase the mortality and disability rates of patients. The recurrence rate after transplantation is relatively high, with a median survival period of 4 to 6 months and a 1-year survival rate of less than 20% (80). For older patients with multiple comorbidities, transplantation-related toxicity is difficult to tolerate, and allo-HSCT is not suitable.

### 5.3 Innovation of diagnostic technology

The long-term use of FLT3 inhibitors such as Sorafenib increases the risk of drug resistance. To efficiently understand the mechanism of drug resistance, more diagnostic techniques need to be combined to e monitor the changes of the disease dynamically and to optimize the treatment plan. In clinical treatment, liquid biopsy techniques have made progress in cell-free DNA (cfDNA) and circulating tumor DNA (ctDNA) in solid oncology (81, 82). Most cfDNA originates from hematopoietic cells (83), with 150–200 base fragments (84). Under different pathological conditions, its size can be affected. cfDNA can reflect the pathological progression process in real time due to its short half-life. At present, the prognosis of ctDNA has been verified in solid tumors and hematological malignancies. In colon cancer, ctDNA is used as an indicator of recurrence to improve the treatment plan (85). Meanwhile, in diffuse large B-cell lymphoma, baseline ctDNA and ctDNA are regarded as independent prognostic indicators (86). In addition, microRNA and cellular protein expression (CD markers) can also be used as independent prognostic indicators for it (87).

### 5.4 The clinical transformation of new targeted drugs

#### 5.4.1 Target D835/F691

Please refer to **Figure 3** for details on the clinical transformation of new targeted drug. Sitravatinib effectively inhibits the phosphorylation of PDGFRα, PDGFRβ, IGF1-R, and c-MET (88), demonstrating antitumor activity in clinical trials for solid tumors (89). Research by Zhang et al. showed that sitravatinib inhibits FLT3 cell proliferation in both MOLM13 xenograft and BaF3 models with various FLT3 mutations (90). Currently, Sitravatinib has demonstrated manageable safety and moderate clinical efficacy in phase 1/1b trials for advanced solid tumors (91). It remains in preclinical development for AML, with its antitumor activity confirmed in AML models. Additionally, it targets the D835/F691 mutation, presenting a potential strategy for overcoming FLT3 inhibitor resistance (90).

#### 5.4.2 FLT3 covalent inhibitor

FF-10101 is a selective FLT3 inhibitor that covalently binds to a specific cysteine residue (such as cys695) in FLT3 kinase domain through a covalent warhead (such as an acrylamide group) in its molecular structure (92). The related research has shown that FF-10101 can reduce an FLT3-ITD mutation with quizartinib-resistant D835Y, Y842C/H, and F691L TKD mutations (92). A phase 1 trial involving R/R AML recently achieved a 10% CR rate and a 12.5% OS rate among 40 participants (93).

#### 5.4.3 New tyrosine kinase inhibitor

Foretinib is a novel tyrosine kinase inhibitor mainly targeting c-MET and VEGFR2 (94). It has demonstrated significant antitumor activity in various solid tumors such as gastric, ovarian, renal cell, liver, and non-small cell cancers. Wang et al. confirmed foretinib’s efficacy against multiple drug-resistant mutations (D835, F961, and Y842 mutations) through *in vitro* and *in vivo* experiments, showing a lower IC50 value compared to other FLT3 inhibitors. It effectively inhibits the proliferation of human AML cell lines (95). In the ongoing Phase II clinical trial of foretinib (GSK1363089) for recurrent or metastatic head and neck squamous cell carcinoma, 50% of patients (7/14) achieved stable disease (SD), while 43% (6/14) demonstrated tumor shrinkage (96). Two patients maintained stable disease progression for ≥13 months, demonstrating the drug’s efficacy and disease stabilization in this patient population. However, there are currently no clinical trials for foretinib in AML.

#### 5.4.4 Nano-loaded combination drugs

1. Sorafenib supports micellar nanocomposites

Sorafenib is utilized for treating FLT3-ITD-positive AML in various contexts (97), but its effectiveness is often limited. Researchers developed a bone marrow-targeted nanomedicine called Sorafenib-Loaded Micellar Nanocomplex (Sora-MN) to address this. This complex self-assembles from sorafenib and HA-EGCG (Epigallocatechin-3-gallate-Hyaluronic acid) in an aqueous environment and is purified via centrifugal filtration. This complex, composed of sorafenib and HA-EGCG, demonstrated improved efficacy compared to free sorafenib by effectively inhibiting the mTOR pathway. Pharmacokinetic studies demonstrated that Sora-MN enhances bone marrow accumulation of AML cells without adverse effects, demonstrating a promising biosafety profile (98).

2. CXCR4-mediated co-delivery of FLT3 and BCL-2 inhibitors.

In acute leukemia, C-X-C Chemokine Receptor Type 4 (CXCR4) is markedly overexpressed and has been shown to correlate positively with resistance and unfavorable survival outcomes (99, 100). Yang et al. employed a combination therapy using the CXCR4-mediated BCL2 inhibitor Venetoclax (VEN) and the FLT3 inhibitor SOR, delivered *in situ* to FLT3-ITD mice via a T22-peptide labeled disulfide crosslinked polymer micelle (TM). Their findings indicated that this approach significantly extended the median survival of MV-4–11 AML mice (101), suggesting targeted nano delivery of these inhibitors may be an effective new strategy for AML treatment.

#### 5.4.5 Small molecule inhibitor combination

Recent investigations have shown a significant increase in SPHK1 expression in FLT3-ITD-positive cells following extended treatment with sorafenib. This finding has been substantiated through Western blot analysis, which confirmed the elevated levels of SPHK1 and S1P (46). Jiang et al., using quantitative PCR, indicated that both SPHK1 and β-catenin are upregulated, offering added protection to these cells. Prior findings have indicated that inhibiting SPHK1 boosts the activation of PP2A and GSK3β, leading to β-catenin degradation in FLT3-ITD AML cells (46). This combined effect improves the efficacy of sorafenib and quizartinib therapies.

#### 5.4.6 Combination with immunotherapeutic agents

CD8+ T cells are essential in fighting infections and tumors. Sorafenib has been proven to enhance interleukin 5 (IL-5) expression in FLT3-ITD and leukemia cells, improving CD8+ T-cell responses and survival in murine models (102). While sorafenib monotherapy reduced IL-15 levels (102), combining T cells with sorafenib increased IL-15 expression. Interferon regulatory factor 7 (IRF7) is crucial for IL-15 transcription (103), but Activating Transcription Factor 4 (ATF4) negatively regulates IRF7, inhibiting IL-15 production. In both mouse and human leukemia cells treated with sorafenib, IRF7 expression increased, while ATF4 levels decreased. However, these changes were not evident in sorafenib-resistant leukemia cells, indicating a mechanism through which sorafenib boosts IL-15 production by repressing ATF4 in FLT3-ITD AML (102).

#### 5.4.7 Gene editing combined therapy

The latest research indicates that by targeting and knocking out FLT3 gene through CRISPR/Cas9 technology, the functional differences of FLT3 in acute myeloid leukemia (AML) can be systematically analyzed. Experiments have demonstrated that FLT3 is dependent on leukemia stem cells (LSCs) that carry internal tandem duplication (ITD) mutations. ITD-mutated AML transplanted tumors disappear within 12 weeks after knockout. Additionally, short-term transplantation reveals that unedited ITD-positive cells eventually eliminate edited cells. However, wild-type LSCs and normal hematopoietic stem cells (HSCs) are not dependent on FLT3, and normal HSCs can still maintain multi-lineage hematopoietic reestablishment ability. Mechanism studies have found that the deletion of FLT3 specifically inhibits the DNA repair and cell cycle checkpoint pathways of ITD-positive LSCs, leading to their proliferation obstruction and increased apoptosis. This achievement has established the feasibility of FLT3 as a precise therapeutic target for AML, providing a theoretical basis for the development of novel therapies for selectively eliminating ITD-mutated LSCs, while avoiding the problem of normal hematopoietic inhibition caused by off-target effects of traditional FLT3 inhibitors (104).

#### 5.4.8 Other therapy

1. Melatonin is applied in combination with sorafenib or alone.

Melatonin (N-acetyl-5-methoxytryptamine) is an antioxidant that protects cells from damage and has anti tumor properties in various cancers, including lung and breast cancer. It enhances the effectiveness of chemotherapy by inhibiting key pathways like PI3K/AKT and NF-κB (105). Emerging studies have indicated that integrating redox-modulating agents like melatonin may enhance conventional chemotherapy regimens’ anti tumor activity and selectivity, minimizing associated toxicities (106). For example, research has shown that melatonin combined with sorafenib synergizes with AML cells (107), highlighting its potential as an adjunctive treatment for hematological malignancies.

2. All-trans retinoic acid works synergistically with FLT3 inhibition.

Retinoic acid (RA) is vital in differentiating HSCs. It has significantly improved cure rates for acute promyelocytic leukemia (APL) through all-trans retinoic acid (ATRA) treatment (108). Retinoic acid (RA), also known as all-trans retinoic acid, is a vital fat-soluble vitamin that maintains normal human metabolism and bodily functions (109). In the early 1960s, ATRA was first used to treat skin diseases. It also involved in the central nervous system, cancer prevention, and metabolic disorders, etc. (110). In 1966, when the Shanghai research team administered ATRA as monotherapy, it achieved an 85% complete remission rate (CR) in acute promyelocytic leukemia (APL) patients (111).

ATRA demonstrates notable efficacy as a monotherapy for AML. Ma et al. (112)have shown that sorafenib and ATRA combination exhibits a synergistic effect, enhancing *in vivo* survival when treating FLT3-mutated AML. This upregulates the apoptotic protein Bcl6, which mitigates the drug’s apoptotic effects. In murine models of AML, concurrent use of ATRA and sorafenib depleted FLT3-ITD leukemia stem cells and prolonged survival, thus significantly reducing recurrence rates in affected patients.

## 6 In conclusion and future perspectives

Sorafenib has demonstrated significant therapeutic potential in treating hepatocellular carcinoma, renal cell carcinoma, and FLT3-mutated acute myeloid leukemia (AML), yet its clinical efficacy is often constrained by drug resistance arising from tumor heterogeneity. The development of resistance is not the result of a single mechanism, but rather a multi-level, overlapping, dynamic evolutionary process. In FLT3-mutated AML, this process involves complex interactions between primary mechanisms (such as inherent survival of tumor stem cells) and secondary mechanisms (including secondary mutations in FLT3 kinase domain under therapeutic stress, activation of alternative signaling pathways like RAS/MAPK, and tumor microenvironmental protection). These interactions collectively lead to off-target effects and disease recurrence.

To systematically unravel this complexity and achieve precise dynamic monitoring of disease progression, integrating multi-omics evidence with cutting-edge technologies is crucial. In AML, next-generation sequencing (NGS) and liquid biopsy—combining dynamic monitoring of mutation profiles in circulating free DNA (cfDNA), cDNA expression changes, and disease-specific microRNAs—form a non-invasive, real-time molecular radar that can detect the emergence of drug-resistant clones before clinical symptoms appear. Building on this, specific omics analyses in AML further reveal the intrinsic drivers of drug resistance. Transcriptomics identifies gene expression signatures associated with drug resistance, such as pathways related to apoptosis evasion and enhanced DNA repair. Epigenomics reveals how DNA methylation and histone modifications reconfigure tumor suppressor genes or activate oncogenes, leading to sustained drug resistance. Proteomics and phosphoproteomics directly map the dynamic reprogramming of FLT3-downstream signaling networks, capturing activity changes in key proteins and providing direct evidence of bypass activation. Single-cell sequencing takes this further by analyzing tumor cell heterogeneity at single-cell resolution, identifying rare subpopulations responsible for drug resistance and their clonal evolution pathways.

In addressing drug-resistant therapeutic approaches, next-generation FLT3 inhibitors (e.g., Gilteritinib, Quinapatinib, FF-10101) demonstrate enhanced antitumor efficacy through optimized molecular structures. However, their safety profile requires careful consideration: common hematological toxicities include grade 3–4 bone marrow suppression (neutropenia, thrombocytopenia), while non-hematological concerns warrant particular attention to elevated liver enzymes (AST/ALT) and potential QTc interval prolongation. These factors require close monitoring and management during clinical use.

Meanwhile, mechanism-based combination therapies demonstrate greater potential. Sorafenib in combination with a nano-delivery system enhances targeted drug accumulation at bone marrow lesions while reducing systemic exposure and associated toxicity. The combination of sorafenib with SPHK1 inhibitors aims to synergistically block sphingolipid metabolic signaling pathways, overcoming metabolic adaptation resistance. Sorafenib combined with immunotherapies (e.g., PD-1/PD-L1 inhibitors) seeks to reverse the immunosuppressive microenvironment while targeting driving signals. Sorafenib, in combination with melatonin, explores enhancing chemotherapy sensitivity and protecting normal tissues by regulating circadian rhythms and oxidative stress.

In summary, the development of resistance to sorafenib in FLT3-mutated AML represents a multidimensional and overlapping biological process involving the genome, signaling networks, and microenvironment. Moving forward, we must deeply integrate next-generation sequencing (NGS) with liquid biopsy-based dynamic monitoring systems into multi-omics precision molecular profiling. Building on this foundation, we need to rigorously evaluate the efficacy and safety of novel inhibitors and combination therapies—particularly their hematological and non-hematological toxicities—to develop truly personalized treatment plans with dynamic adjustments. This approach will ultimately enable systematic solutions to overcome drug resistance challenges.

## Glossary

AML: Acute myeloid leukemia
FLT3-TKIs: FLT3 tyrosine kinase inhibitors
FLT3: FMS-like tyrosine kinase 3
FLT3-ITD: FLT3 internal tandem duplication
FLT3-TKD: FLT3 tyrosine kinase domain
OS: overall survival
DFS: disease-free survival
HSCs: hematopoietic stem cells
FLT3L: FLT3 ligand
R/R: relapsed/refractory
A-loop: activation loop
IG-like-domain: IG-like domain-containing proteins
JM domain: juxtamembrane domain
TKD1: tyrosine kinase domain 1
TKD2: tyrosine kinase domain 2
JAK: janus kinase
STAT5a: Signal Transducer and Activator of Transcription 5A
GRB2: Growth Factor Receptor Bound Protein 2
RAS: oncogene
RAF: Raf-1 Proto-Oncogene, Serine/Threonine Kinase
ERK1/2: Extracellular regulated protein kinases 1/2
SHC: Src homolog and collagen homolog
PI3K: Phosphatidylinositol 3-kinase
AKT: Protein kinase B
mTOR: mammalian target of rapamycin
FOXO: Forkhead Box O1
S6K: Ribosomal Protein S6 Kinase B1
p36: Phospho-S6 Ribosomal Protein
EFS: event-free survival
RFS: relapse-free survival
HAM: hypomethylating agents
CLAG-M: granulocyte colony-stimulating factor, and mitoxantrone
KIT: The stem-cell factor receptor (CD117)
PKC: Protein kinase C
FLT3-ITD-positive: FLT3-ITD+
YAP1: Yes-associated protein 1
HDAC: histone deacetylase
HDAC3: histone deacetylase 3
HDAC10: histone deacetylase 10
H3K27: histone H3 lysine 27
PPP: pentose phosphate pathway
R5P: ribulose-5-phosphate
γ-Glu-Cys: γ-glutathione-cysteine
SPHKs: Sphingosine kinases
S1P: sphingosine-1-phosphate
β-catenin: Catenin beta-1
RCD: Regulatory cell death
ROS: reactive oxygen species
GPX4: glutathione peroxidase 4
GSH: synthesis of glutathione
BMAL1: Basic Helix-Loop-Helix ARNT Like 1
LC3B-II/I: Microtubule-associated protein 1 light chain 3
ATG5: Autophagy Related 5
p62: Sequestosome 1
GSK3β: glycogen synthase kinase 3beta
STAT3: signal transducer and activator of transcription 3
BMX: Bone marrow kinase on chromosome X
HIFs: hypoxia-inducible factors
VHL: Von Hippel-Lindau
BMM: bone marrow microenvironment
HSCs: Hematopoietic stem cells
FGF2: fibroblast growth factor 2
FGFR: FGF receptor
CYPs: cytochrome P450 enzymes
LSP1: Lymphocyte-specific protein 1
CD8: Cytotoxic T lymphocyte
allo-HSCT: Allogeneic hematopoietic stem cell transplantation
GVL: graft-versus-leukemia
GVHD: graft-versus-host disease
cfDNA: cell-free DNA
ctDNA: circulating tumor DNA
FDA: Food and Drug Administration
AXL: AXL Receptor Tyrosine Kinase
CR/CRi: Complete Remission/Complete Remission with Incomplete Hematologic Recovery
Sora-MN: Sorafenib- Loaded Micellar Nanocomplex
HA-EGCG: Epigallocatechin-3-gallate-Hyaluronic acid
TM: T22-peptide labeled disulfide
IRF7: Interferon regulatory factor 7
LSCs: leukemia stem cells
HSCs: hematopoietic stem cells
Melatonin: N-acetyl-5-methoxytryptamine
RA: Retinoic acid
APL: acute promyelocytic leukemia
ATRA: all-trans retinoic acid
CXCR4: C-X-C chemokine receptor type 4
HAM: hypomethylating agents
NPM1: Nucleophosmin 1
FMS: FMS-like tyrosine kinase 3
PDGFR: Platelet-derived growth factor receptor
KIT: the stem-cell factor receptor, CD117
SCT: stem cell transplantation
CR: Complete response
SPHK1: Sphingosine kinase 1
SPHK2: Sphingosine kinase 2
IL-5: interleukin 5
